# Comparison of Wood-Based Biocomposites with Polylactic Acid (PLA) Density Profiles by Desaturation and X-ray Spectrum Methods

**DOI:** 10.3390/ma16175729

**Published:** 2023-08-22

**Authors:** Seweryn Pycka, Kamil Roman

**Affiliations:** 1 Faculty of Wood Technology, Warsaw University of Life Sciences-SGGW, 166 Nowoursynowska St., 02-787 Warsaw, Poland; s190114@sggw.edu.pl; 2 Institute of Wood Sciences and Furniture, Warsaw University of Life Sciences, 166 Nowoursynowska St., 02-787 Warsaw, Poland

**Keywords:** wood–plastic composites, biocomposite, polylactide (polylactic acid), forest residues

## Abstract

Wood–plastic composites (WPCs) represent composite materials that employ shredded wood combined with a thermoplastic substance, such as polylactic acid (PLA), to establish structural cohesion within the product profile. This amalgamation of materials results in a robust structure designed to fulfill specialized roles under the influence of pressure and temperature. Given the nature of the constituent materials, the resultant product can be classified as a biocomposite. The creation of such biocomposites entails a rigorous process necessitating the fine-tuning of specific parameters and suitable technologies. The foundational materials employed in this process must be both natural and biodegradable. However, it is noteworthy that natural components like fibers exhibit anisotropic behavior, wherein their mechanical attributes are contingent on the direction of the applied force. Consequently, predicting their performance during biocomposite production proves challenging. The principal objective of this study was to conduct a comparative analysis of wood-based composites incorporating PLA thermoplastic binding agents. The intention was to discern variations in density profiles arising from distinct measurement methodologies. Two measurement methods were used for the measurement: X-ray and spectrum desaturation. Additionally, the study sought to investigate the impact of introducing PLA additives at 25% and 50% concentrations on the fabrication of WPC from wood chips. The properties of these composites were assessed by considering the inherent traits of the composite materials.

## 1. Introduction

The modern approach to wood construction requires use of dedicated materials that should be characterized by specialized properties. The solution, in this case, can be wood–plastic composite (WPC) production [1], with biodegradable parameters named biocomposites. The term “biocomposite” refers to a composite material in which at least one of its components is of biological origin, deriving from living organisms or biodegradable materials. Biocomposites utilize natural materials as reinforcement components or as matrices, along with other biodegradable polymers as binders.

The primary goal in creating biocomposites is often to achieve environmental benefits, such as reducing the consumption of non-renewable resources and decreasing greenhouse gas emissions by substituting traditional synthetic materials. Examples of biocomposites include wood–plastic composites (WPCs), where wood serves as the reinforcement component, a thermoplastic polymer acts as the binder, and plant-fiber composites, like hemp, jute, or flax, are used as reinforcement in biodegradable polymers.

Biocomposites find applications across various industries, including the automotive sector, construction, packaging, and the production of consumer goods, where sustainable and environmentally friendly materials are becoming increasingly sought after. The WPC biocomposites [2] are opening new ways of applications in many areas, including furniture, construction, and packaging. Wood-based composites are also suitable for outdoor applications that require durability and low maintenance. Compared to traditional wood products, WPC products may not feel as natural [3]. After extruding the polymer, it can be shaped into various shapes, such as boards, profiles, or pellets. The new component should characterize with high durability and low maintenance structure. They should also be fit for the purpose that they were created for. To produce a biocomposite with the desired mechanical properties and resistance to external factors, the right ingredients and technological processes must be selected. Inhomogeneity is the feature that adversely affects the technical value but is an advantage biologically as well, so the way the raw material is used is important in this regard [4].

The production of WPC consists of the blending process to create a suitable product. In biocomposites, natural fibers from wood, hemp, or fast-growing natural fibers, are used to form composite materials. They are also made up of a biodegradable polymer matrix, which can include polylactic acid (PLA) or a polymer based on starch [5]. These materials can be combined into a lightweight, strong, and environmentally friendly material that can be used in various applications. The use of biocomposites has several advantages over the use of traditional composite materials [6]. Compared with traditional wood products, the materials that result from the process have increased resistance to moisture [7,8]. Using natural fibers has other advantages over synthetic fibers, such as the fact that they are renewable and biodegradable [9]. The mechanical properties of natural fibers are superior to those of synthetic fibers, such as increased stiffness, strength, and impact resistance compared to synthetic fibers [10]. 

There are a number of benefits of biocomposites over traditional composites, such as lower production costs due to the cheaper cost of raw materials This article expands information about the basic physical and mechanical properties of PLA that may be beneficial for the development of composites and biocomposites [11,12].

Assumptions about biocomposites include easy maintenance and recyclability at the end of life [13]. The disadvantages of biocomposites include lower heat resistance than traditional composites and the possibility that they are more sensitive to moisture [14]. It is also important to note that the properties of biocomposites are highly dependent on the type of natural fibers and polymer matrix that are included in the formulation [15]. The use of biocomposites is an innovative and sustainable alternative to the traditional use of composite materials [16]. The potential for these materials to be widely used in a diverse range of industries is highly promising with further research and development. The development of research methods can enable the properties of produced biocomposite to be more controlled.

This article aims to fill the existing gap in the literature regarding the combined use of PLA and Scots pine (*Pinus sylvestris* L.) wood in composite production. The selected and utilized Scots pine (*Pinus sylvestris* L.) wood material was intentionally chosen due to its predominant presence in the region. Despite existing research on PLA and wood composites, there is limited literature on the joint utilization of PLA and Scots pine (*Pinus sylvestris* L.) wood fibers. The existing body of work does not provide sufficiently detailed information about the influence of different process parameters and the proportion of PLA to Scots pine (*Pinus sylvestris* L.) wood fibers on the mechanical, thermal, and ecological properties of such composites. This manuscript aims to identify and address this knowledge gap, leading to a better understanding of the potential of utilizing PLA and Scots pine (*Pinus sylvestris* L.) wood fibers as composite materials and their applicability in the production of durable and environmentally friendly products. The hemicelluloses of softwoods (hexoses) are less unstable than those of hardwoods (pentoses) because of the different composition [17].

## 2. Materials and Methods

### 2.1. Material

The material utilized for the conducted studies was a composite comprising forest residues from pine wood (*Pinus sylvestris* L.) and polylactic acid (PLA), serving as a thermoplastic binder. Pine shavings had a thickness ranging from 0.5 mm to 1 mm. Their length, on the other hand, ranged from 1 mm to 2 mm. This composite is formulated by combining wood fibers with a thermoplastic polymer to yield a cohesive material [18].

Common pine wood is widely used in construction, furniture making, carpentry, and many other fields. In the described case, the common pine wood was felled at the age of 90 years. It was a mature tree. After felling, the wood underwent a drying process, resulting in its moisture content being reduced to 12%. The appropriate moisture level is essential to prevent deformations and cracks in the wood after processing. The average true density of the wood is around 470 kg/m^3^.

In the context of this investigation, poly (lactic acid) (PLA) was chosen as the thermoplastic component for the produced biocomposite. Notably, the majority of plant-derived thermoplastics are derived from sources like corn and potatoes, imbuing them with enhanced environmental friendliness [19]. The material itself exhibits a notable degree of elasticity and plasticity, with these properties increasing as melt flow elevates [20], achieved at temperatures ranging from 190 to 230 °C.

Polylactic acid, also known as PLA, is a biodegradable synthetic polymer that is gaining popularity due to its positive environmental properties and wide range of applications. Its chemical structure is based on lactic acid, a naturally occurring organic compound. PLA is synthesized through the polymerization of lactic acid or its lactone. PLA molecules consist of polymer chains composed of lactic acid monomer units. This monomer has two enantiomeric isomers, D-lactide and L-lactide, which give rise to corresponding isomers of the PLA polymer. PLA is a unique example of a chiral polymer, meaning it has an asymmetric arrangement of atoms, affecting its physical and chemical properties. It exhibits high crystallinity, which impacts its strength and stiffness. PLA can have varied properties depending on the isomer ratio and degree of polymerization. It is biocompatible and biodegradable, making it an attractive material for medical applications, eco-friendly packaging, and the textile industry. Due to its chemical structure, PLA has the potential to replace traditional synthetic materials that are less environmentally friendly. Ongoing research into its improvement and applications aims to utilize PLA as a sustainable solution across various industries and daily life.

From a commercial standpoint, this composite can be readily sourced from establishments specializing in 3D printing components and plastics [21,22], facilitating easy acquisition. An advantage of the material’s formation process lies in its inherent eco-friendliness [23]. Moreover, it is essential to underline that all the work was conducted at room temperature, which was maintained at 23 degrees Celsius throughout the study.

This study shows that composites made of polylactic acid (PLA) have great potential to replace traditional synthetic materials that cannot decompose or be renewed. Using PLA-based biocomposites is a fantastic way to produce sustainable products [24].

To conduct the study, the wood raw material and PLA required pre-treatment. Notably, the entire material utilized in the research was subjected to mixing via a drum mixer. The employed material was shredded to a particle size fraction of 1 mm, specifically tailored for the study’s objectives. Adhering to standards EN ISO 15149-1:2011 [25] and EN ISO 15149-2:2011 [26], the prescribed tests were performed.

During the 120 s testing process, fractions ranging from 0 to 1 mm were separated from the test material. This shredding protocol was consistently applied to both the wood and PLA materials employed within the study. Following fragmentation, the resulting material was blended based on its weight post-shredding. This meticulous material preparation enabled the creation of a composite mix comprising 25% and 50% PLA combined with wood, thereby establishing a foundation for subsequent research endeavors.

During the studies, the sample hygroscopicity parameter was also taken into account by measurement of moisture content. Based on the guidelines, the wood material was kept at a constant moisture level [26]. Shredded prepared mixture samples were tested for moisture content in order to ensure that the moisture content of the prepared material was controlled carefully before it was compacted. During the testing process, a Radwag WS30 weighing dryer was used in order to perform the weighting test. In addition to the physical parameters, the ash content may also have affected the measured density profile [5]. Detailed information about the mineral saturation of the raw material can be obtained from its ash content [27]. According to the literature, the suggested moisture content in the compacted biocomposites raw material should be about 12% [28].

According to similar studies [26], the percent content of mineral compounds and moisture content of the measured raw material for the native samples were measured. It turns out that the combination of pine wood (*Pinus sylvestris* L.) in a biocomposite with PLA does not have statistically significant differences. Ash content for pure PLA was roughly 1.4%, and the same value was also found for PLA mixtures of 50%, 25%, and pure milled-wood residues [29].

### 2.2. Compaction in the Heating Plates

**Figure 1:**
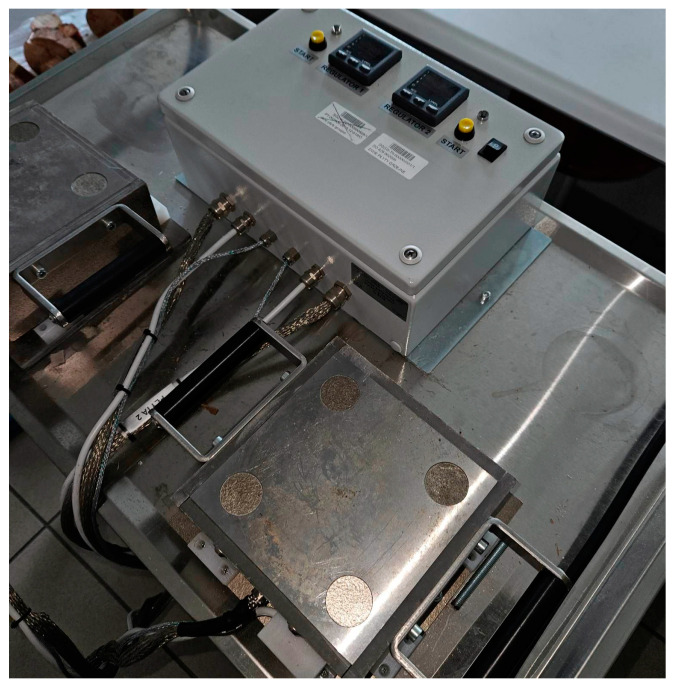
The compaction heating plates.

In order to carry out the planned tests, it was necessary to use a heating plate. In order to perform the experiments, an appropriate test measuring stand was constructed to prepare the specimens for future testing. In the first step, the prepared raw material sample before compacting was pretreated in the oven heated up to 230 °C for 10 min. The second step was to compact the prepared mixture between the heating plates at the desired temperature after pretreatment. It was necessary to maintain a constant temperature provided by the controller for the heater during the compacting process. The required temperature in this case obtained at 160 °C and the necessary time obtain in about 10 min. The compaction heating plates are presented in Figure 1.

Static compression with the strength to compact the sample was performed. If the structure of the produced sample did not meet the required quality, alternative force values were used. The compaction process was applied to samples with a volume of 9.5 cm^3^. The constant parameters were the length of 500 mm and the width of 500 mm for the compact form. The compaction heating plate worked with a maximum compaction strength of 625 N.

### 2.3. Microscope Aperture

The analysis of biocomposite samples under a microscope can reveal valuable information about their composition, structure, and properties [22]. These materials can be developed and optimized for various applications in fields such as environmental science using that information. Optical analysis was conducted by scanning the microscope with a Nikon SMZ 1500 and analyzing the data with a computer program. There is a 15:1 zoom range on this stereoscopic microscope, which includes zooms that range from 0.75 to 112.5 magnification. In this way, sample visualizations can range from macro views to micro-views at high magnifications. This microscope is equipped with an optical system that is capable of covering the full range of aberrations in regard to chromatic aberration and planarity. In addition, it has a 150-watt halogen illuminator for its illumination.

**Figure 2:**
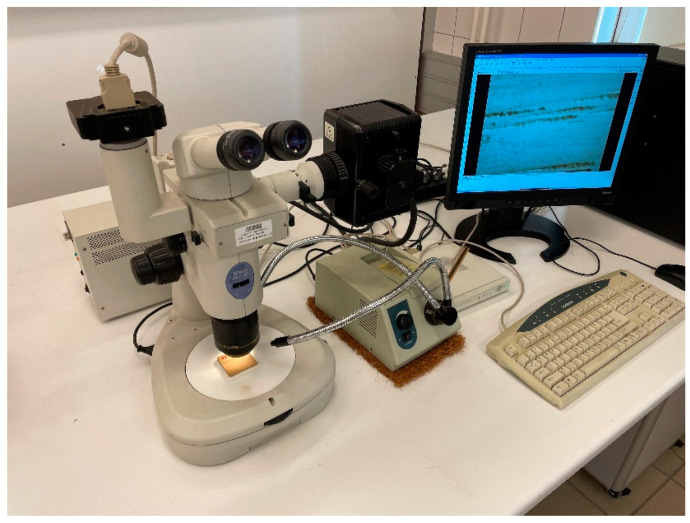
The Nikon SMZ 1500 microscope set.

The Nikon SMZ 1500 is a high-performance stereoscopic microscope that provides clear and detailed images at high magnification. The microscope has a high-quality optical system that provides excellent resolution and contrast. The Nikon SMZ 1500 is an advanced camera, featuring advanced features that make it ideal for research and laboratory use. Sample preparation and analysis are made flexible by the range of lighting options, which includes both reflected and transmitted illumination. This methodology was carried out using the assumptions and featured advanced controls and an intuitive interface for exporting images and applying screen filters. The Nikon SMZ 1500 microscope set is presented in Figure 2.

Microscope research offers many opportunities for scientists and researchers in a wide range of fields. The microscope in research can observe and analyze samples at a microscopic level. Using the microscope aperture together with the picture desaturation method, researchers are able to study the properties and behaviors of materials. Microscope research can therefore provide unique insights into a wide range of phenomena, from solid structures to materials and their surface properties. In addition to providing detailed observations, microscope research can also generate quantitative data to model and predict behavior. In the future, the gathered data can be used to test hypotheses and develop more accurate theories. Imaging techniques allow examining samples with a wide range of microscopic features. The possibilities that can be explored through microscope research are almost limitless as technology continues to advance in interdisciplinary fields.

### 2.4. Picture Desaturation

**Figure 3:**
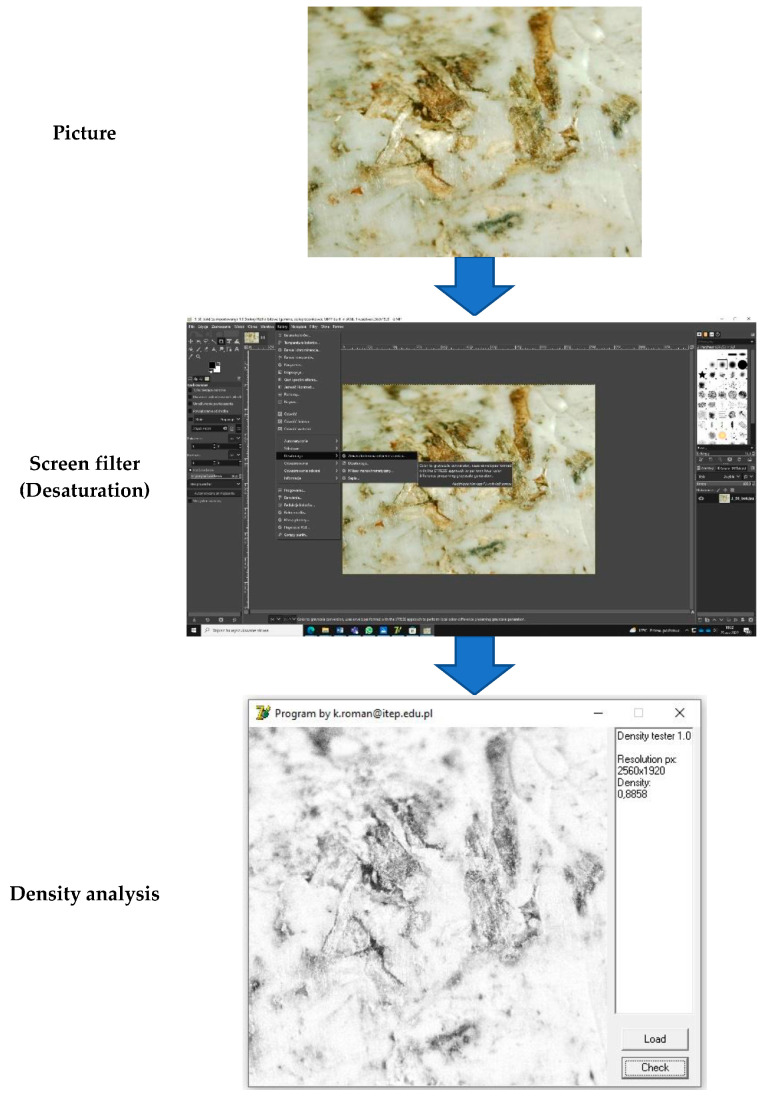
Schematic of the working algorithm for screen desaturation.

The process of converting a source image into a final image form was performed based on a specially prepared computer program. The process of transforming an image involves permanent changes in colors by converting RGB images into grayscale images. Changing a color image into grayscale causes irreversible data loss, making it impossible to recover the RGB mode. The GIMP (GNU Image Manipulation Program) v. 2.20.32 software program was used to analyze the images. Through the use of special sophisticated tools, a filter is used in order to desaturate the picture. An algorithm in a specially designed program analyzes pixel colors by counting gray shades. Delphi was used as the programming language to develop the interface between the computer program and the user. An example of a working algorithm for screen desaturation is presented in Figure 3.

The information about color saturation in the image is reduced when it is desaturated to the grayscale mode by reducing its saturation level [26]. Grayscale reduction is the process of removing the hue and saturation information from the pixels of a picture. The information saved with the image is only a measure of the luminance of a particular image. In order to further analyze an image, a program with specific instructions is used to count the intensity of grayscale pixels. Computer programs prepare an algorithm to count and separate pixels based on grayscale. The combination of the obtained data allows the estimation of the amount of black-to-white in an image.

### 2.5. Density Profile

**Figure 4:**
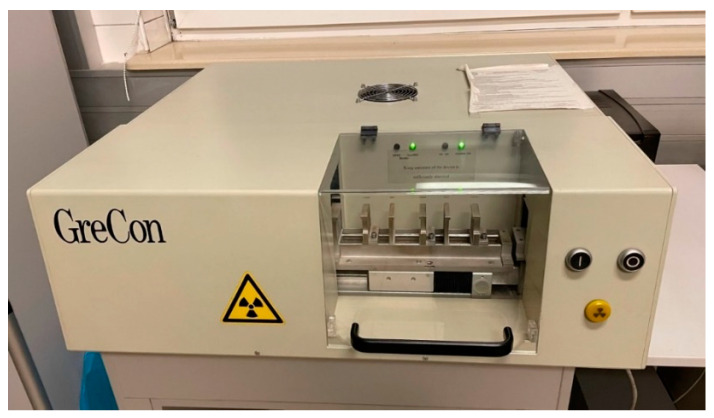
Test stands for the X-ray density profile measured by GreCon.

In order to perform the biocomposite structure tests, the density profile analysis was one of the most crucial research elements. The analysis of the biocomposite density profile was tested in two different PLA mixture proportions. The analysis allows an understanding of how the percentage of PLA in the value of 25 and 50% affects the structure and density of the material at different test points of the sample. The test allows determining the size of changes in the biocomposite structure depending on the intensity of the assumed percentage of PLA. According to the methodology, the measured samples required technological preparation. The sample profile must be in a dimension not exceeding 500 × 500 mm in width and height. The dimensional range ensures the proper measurement in GreCon measurement equipment. The prepared sample was measured with a caliper, controlling the dimensions of width, height, and thickness in accordance with the measurement equipment recommendations. The density profile of the prepared mixtures was measured at a speed 0.05 mm⋅s^−1^. The GreCon measurement equipment for the density profile test is presented in Figure 4.

The biocomposite density sample profiles were measured by X-ray densitometry using a GreCon device. The tested biocomposite samples are placed on a frame with the tray in the device. During measurement, the integrated measuring tray moves along the sample measuring the density of the biocomposite at specific points. The mounted samples were subjected to X-ray densitometry, where the X-ray radiation penetrated the analyzed material. The X-ray waves are emitted from the measuring head and detected with an integrated detector. The generated data were prepared in a spreadsheet and could be transformed into a graph illustrating the sample density profile. The radiographs and density distributions along the length of the sample cross-section are the X-ray densitometry results. It is possible to read the basic statistical data about the density profile in the analyzed area from the generated program datasheet aside from the density profile. As a result of the obtained measurement data, it is possible to digitally characterize and compare the density profile of biocomposites that have been blended with 25% and 50% PLA. This measurement technique has the advantage of being a non-destructive test for the behavior and properties characteristics of the tested material.

### 2.6. Statistical Processing of Results

This study was statistically analyzed using the ANOVA variance [26] as a method for analyzing the results. The method allows for the determination of the influence of wood-based composites that contain pine (*Pinus sylvestris* L.) shredded material with PLA thermoplastic percent material mixture according to density profiles by desaturation and X-ray methods. The statistical analysis was conducted with the assumption that other factors remained invariant (i.e., soli paribus) throughout the process.

## 3. Results

### 3.1. Material Properties

**Figure 5:**
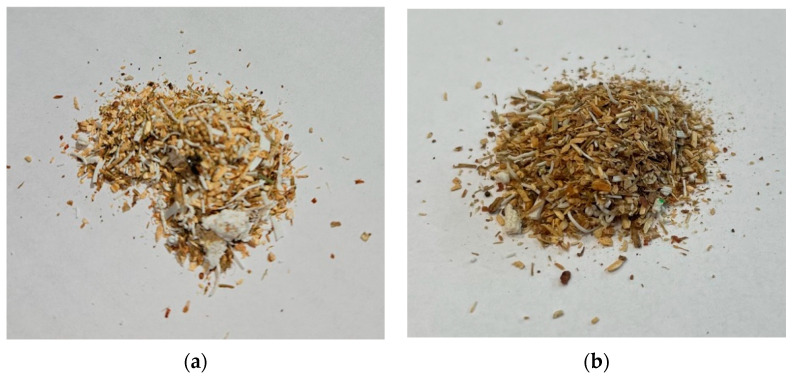
The sample of prepared row material: (**a**) 50% PLA share; (**b**) 25% PLA share.

During the research, wood residues and thermoplastic material were combined with the specified PLA percent content. The wood material in the mixture exhibited anisotropic characteristics, which were caused by its natural structure. Among all wood properties, hygroscopicity is primary because it determines the wood’s moisture content. Moisture changes cause wood to shrink or swell, altering its dimensions. The moisture content has a significant impact on the level of resistance and can be determined based on the moisture content measurement. The moisture content of the wood was determined to be 12%. There are many properties of composites that are affected by moisture, such as their mechanical, thermal, and acoustic properties. The sample of prepared row material composite for 50 and 25% is presented in Figure 5.

The prepared material was pretreated at a high temperature to melt PLA as a thermoplastic bond in produced biocomposite [28]. After that, the prepared dose of pretreated material was ready for future compacting in the heating plates. Compaction of the prepared material was performed on special heating plates that were prepared in advance. According to the guidelines, heating plates with the temperature set were forced with static strength. Materials from the prepared mixtures were compressed at a specified ratio in order to prepare them. In the test specimens, there were two proportions of PLA that were used to prepare the test specimens, 50% and 25% PLA. The prepared samples allowed for the characterization of the PLA density profiles in the wood-based composites analyzed by desaturation and X-rays. The mean density compacted with force 600 N, according to the proportion of PLA content for 50% was 554.59 kg·m^−3^, and for 25%, it was 569.44 kg·m^−3^.

### 3.2. Optical Solid Structure Analyses 

#### 3.2.1. Microscopy Optical Analyses 

**Figure 6:**
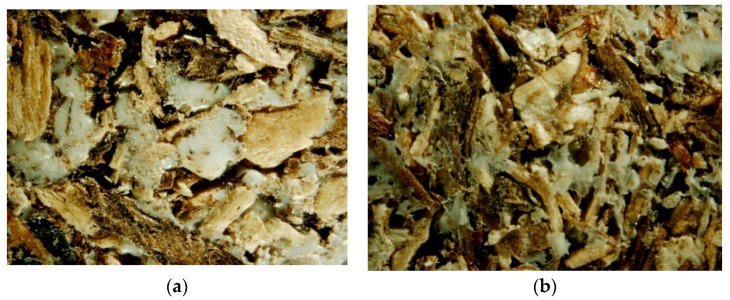
The front view of the profile sample: (**a**) 50% PLA share; (**b**) 25% PLA share.

The PLA biocomposite samples in interdisciplinary research are analyzed to understand their composition, structure, and properties. In order to obtain images with a highly focused zoom for further desaturation activities, the prepared biocomposite samples were examined under the microscope. The biocomposite material was made by combining shredded pine wood (*Pinus sylvestris* L.) with 25 and 50% PLA in mass participation. The samples were analyzed in the front view of the profile. According to the PLA content in the samples, the structure varied according to their initial composition. The front view of the profile samples of 50% and 25% PLA shares is presented in Figure 6.

**Figure 7:**
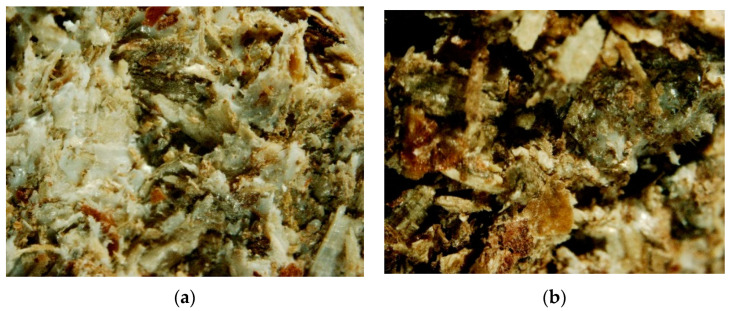
The inside view of the profile sample: (**a**) 50% PLA share; (**b**) 25% PLA share.

Microscopic characterization was intended to provide a general overview of the fraction composition of the biocomposite profile sample with PLA 50 and 25%. The PLA thermocomposite was brought to a semi-liquid state under the influence of temperature, which means that the structure of the biocomposite had a cohesive bond; i.e., PLA had more contact with the wood by wrapping it with its structure. The pictures show that the sample is darker because less PLA was used. The surface of the sample with a higher wood chip content of 25% PLA appears darker and more organoleptically rough. A thin layer of light mass on the surface is a layer of PLA on top. Thermoplastic fibers can be seen more clearly because they arrive in a thicker mass with a white color. Wood chips appear more structured and thickened due to the consistency effect. A smooth texture can be seen on the surface of this object. The sample was additionally examined inside the structure by cutting it into two halves for comparison purposes with the front sample profile view. The edges of both parts were analyzed under a microscope for future computer program analysis. The inside view of the profile sample of 50% and 25% PLA share is presented in Figure 7.

#### 3.2.2. Computer Program Analyses

Microscopically analyzed results of the front sample structure were digitalized by preparing a 2560 × 1920 pixel image. Image desaturation was achieved by applying a special filter that created a gray shade. It was necessary to use a specially prepared program in order to further characterize the image. The computer program used an algorithm to compare grayscale shade intensity in horizontal and vertical loops. Based on the density of the analyzed image, the program calculated a percentage characterization of grayscale share. According to its original purpose, the program determines the density based on practical analysis of X-rays. Similar to X-ray spectrum analysis, the program used white as “1” and black as “0”. In an image being analyzed, these values relate to the proportion of white to black colors.

**Figure 8:**
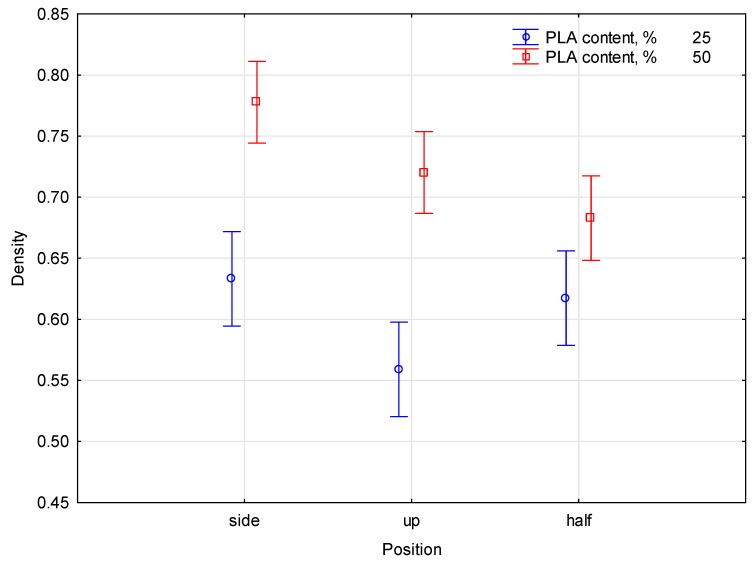
The comparison of the front and inside views of the profile samples.

The results of the analysis were statistically analyzed to determine whether the program was effective in determining PLA percentages from images. The front and inside views of the profile samples were considered. The assumption of the analysis was to determine the PLA percentage from the desaturated image. The proportion of PLA per factor characterizing gray tone density was compared using a one-way ANOVA variance analysis. In order to analyze the image statistically, the image was read and digitalized by a computer program. This diagram illustrates the variation of cases and therefore, the possibility of significant differences between the measured parameters. The analysis of the impact of PLA percentage on the factor associated with grayscale color density is presented in Figure 8. 

In order to determine the significance level effect of the coefficient used to describe the gray shades of color, the density ratio of the PLA content was measured. It was noticed that the significance level of analysis of the comparison of the side, front, and inside views of the profile of the measured samples was less than 0.05. According to the rule to specify statistical effect in terms of PLA content and scanning position of the homogeneous groups with the density, statistical values were calculated. The selected parameters were divided into homogeneous groups by Duncan’s test during statistical analysis. Homogeneous groups determining the affiliation of individual blends with PLA content with respect to the density ratio are summarized in Table 1.

The average density of the tested samples with PLA 50% was 0.726, and for PLA 25% the average density was 0.603. The table shows the density values for different sample positions and different PLA contents.

For samples containing 50% PLA, the following density values were found. Top position: 0.720 (marked as d). Halfway position: 0.683 (marked as c, d). Side position: 0.777 (marked as e). For samples containing 25% PLA, the following density values were found. Top position: 0.559 (marked as a). Halfway position: 0.617 (marked as b). Side position: 0.633 (marked as b, c). Values marked with the letters a, b, c, d indicate belonging to homogeneous groups. On the basis of the statistical analysis performed for the density of the samples, homogeneous groups were determined in which the samples had similar density values.

These conclusions suggest that the mixture of raw materials affects the density of the biocomposite, and that different sample positions and PLA content may lead to differences in density. The division into homogeneous groups enables a better understanding of the interrelationships between the composition of raw materials and the density of the biocomposite. In light of statistical analysis, the difference between PLA content percentages of 50 and 25% was possible. There was a significant impact on the factor defining color density from grayscale tones as a result of this change. In post hoc statistical analyses, it was evident that the measured parameters differed significantly from one another. The decomposition of effective hypotheses created interaction effects where vertical bars meant 0.95. An analysis of the results showed that the *p*-value was equal to 0.0244, making the result significant. The empirical statistic for the study was *F*(2, 77) = 3.8962. This shows a significant difference in the biocomposite material. The values of the results for individual materials, i.e., for 25% and 50%, differed on average by 0.1. This shows that the material has become denser.

### 3.3. Density Profile Structure Analyses 

The density profile of the biocomposite prepared from the mixtures was computed using an X-ray scanner to obtain the density profile of the structure. The study involved the use of a rapid and accurate tool that enabled the characterization of the density profile along the entire length of the sample. An X-ray image allows for noninvasive and accurate measurement of the density of a biocomposite that has been analyzed. Study results were compared between mixtures containing 50% PLA and 25% PLA, which took into account the modified structure of the biocomposite. By measuring the structures and densities of the biocomposite in response to PLA additions, it was possible to accurately determine if a change in internal structure or density occurred. The effect of this process on the material properties of the biocomposite could then be characterized. The biocomposite density profile samples with 50 and 25% PLA content are presented in Table 2. There was an average density of 554.49 kg/m^3^ for the tested samples that contained 50% PLA. For PLA 25%, there was an average density of 569.44 kg/m^3^ in terms of density analysis. Among other reasons, this was due to the fact that the material used in the test was capable of precise cutting. For samples containing 50% PLA, the following was found: Sample I had a height of 52.04 mm, a width of 51.42 mm, a thickness of 7.13 mm, a mass of 10.115 g, and an average density of 530.16 kg/m^3^. Sample II had a height of 50.54 mm, a width of 50.95 mm, a thickness of 7.32 mm, a mass of 11.828 g, and an average density of 627.51 kg/m^3^. Sample III had a height of 51.30 mm, a width of 50.83 mm, a thickness of 7.21 mm, a mass of 9.515 g, and an average density of 506.10 kg/m^3^.

For samples containing 25% PLA, the following was found: Sample I had a height of 49.32 mm, a width of 51.16 mm, a thickness of 7.12 mm, a mass of 10.402 g, and an average density of 579.01 kg/m^3^. Sample II had a height of 50.11 mm, a width of 52.41 mm, a thickness of 7.01 mm, a mass of 10.172 g, and an average density of 552.52 kg/m^3^. Sample III had a height of 49.2 mm, a width of 50.12 mm, a thickness of 7.29 mm, a mass of 10.369 g, and an average density of 576.81 kg/m^3^. These conclusions suggest that the content of PLA affects the density of the biocomposite. Samples containing 50% PLA had an average lower density (530.16 kg/m^3^) compared to samples containing 25% PLA (average 579.01 kg/m^3^). At the same time, samples with different PLA content also differed in size and weight. Increasing the PLA content may lead to changes in the structure and density of the biocomposite. In order to gain a comprehensive understanding of the structure of a biocomposite, a detailed analysis of the density profile was performed with high accuracy. This head displacement along the measured profile’s thickness was 0.02 mm.

This analysis of density profiles is particularly relevant where modified wood–plastic composite materials are being used in weather conditions with variable humidity and temperature levels. Having all this information on hand enables us to check if there are deviations from the norm, which is very helpful. According to the assumptions used in the density profile analysis, there were areas characterized by a lower density that were more susceptible to changes and the impact they would have on the environment. Research of this type enables a more accurate determination of biocomposite density and enables a more individualized application of the material. Density deviations were not observed during the X-ray examination over the width of the spectrum. Which allows us to obtain the best fractional density information. Density profile analysis is particularly important for modified wood–plastic composite materials that are used in atmospheric conditions with varying levels of humidity and temperature. Having this information allows you to check if there are deviations from the norm, which is very helpful.

**Figure 9:**
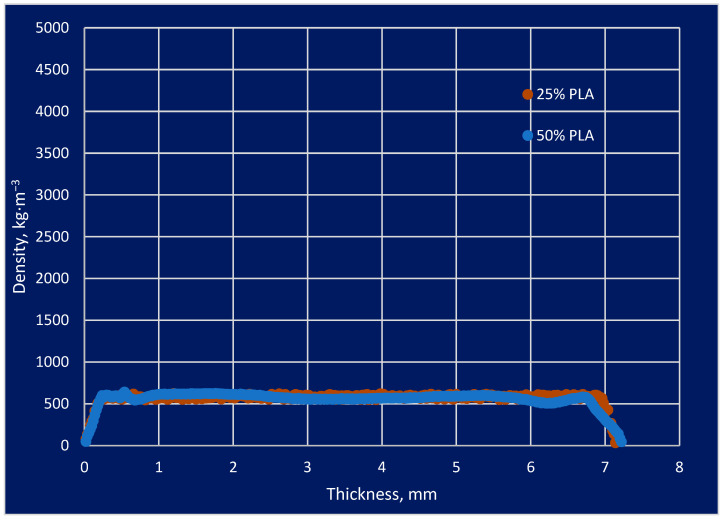
The density profile of the biocomposite sample.

This type of research allows for a more accurate determination of the density of the biocomposite and allows for a more personalized use of the material. Density deviations were not observed over the entire width range during the X-ray examination, which enables the best fractional density information to be obtained. These conclusions suggest that the analysis of density profiles is an important tool in assessing the properties of biocomposites in changing weather conditions. It allows you to monitor possible deviations from the norm and identify areas of lower density that may be more susceptible to changes. This is important for the optimal use of biocomposites in various applications. In addition, the X-ray method provides accurate density information over a wide range, which is important for precise material characterization. The density profile of the biocomposite sample is presented in Figure 9.

According to Figure 9, the density was not equal throughout the thickness of the sample; it can be seen that the graph becomes linear and stays at a density of approximately 500 kg/m^3^ from a certain thickness in the range of 0.3–7 mm. Before this interval, the graph increases linearly, and after the interval, it decreases linearly to 0. The boundaries of the plot for both PLA samples have an upward curve. This is due to the technological process of cutting the samples.

**Figure 10:**
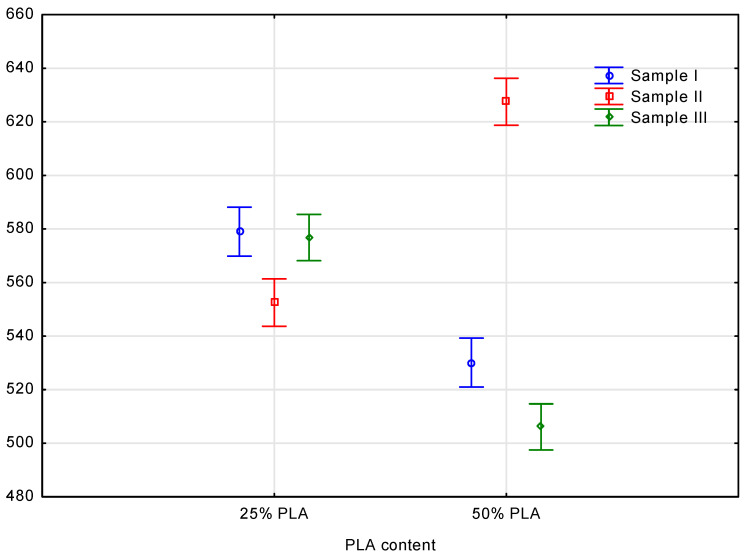
The comparison of the density profile of the thickness profile samples.

In order to determine the difference in density profiles between measurement samples with individual PLA content, analysis results were statistically analyzed. A sample prepared to contain 25% PLA content and a sample prepared to contain 50% PLA content were examined. Based on the X-ray image density profile, it was assumed that the PLA percentage could be calculated. Using a one-way ANOVA and variance analysis to compare the proportion of PLA per factor characterizing the density profile, the proportion of PLA was determined. Using a computer program, a statistical analysis was carried out on the statistical value. In the following diagram, we can see the variation in cases and the possibility that there may be significant differences in the measured parameters between the two groups. A study was carried out to analyze the impact of PLA percentage on the density profile associated with PLA content. The comparison of the density profile of the thickness profile samples is presented in Figure 10.

As a result of statistical analysis, an X-ray spectrum density distribution can be used as a basis for characterizing measurement results in the context of density profiles. The differences between PLA content percentages of 50 and 25% were possible based on the statistical analysis of the data. There is a possibility that the measured parameters of several cases might differ significantly from one another, as shown in the diagram below. All measured parameters showed similarities in statistical analysis based on measurements. It was clear from the outcome of this change that there was a significant impact on the factors that define PLA content as a result of this change. Statistical analysis performed post hoc of the measured parameters revealed that they differed significantly from one another when the results were compared. It was found that there were interaction effects following the decomposition of effective hypotheses in which vertical bars represented 0.95. Based on the analysis of the results, it was found that the *p*-value was lower than 0.05, indicating that the results were significant. Specifically, the empirical statistic calculated for the study is *F*(3, 714) = 946.20, where Wilks Lambda is set to 0.20988.

### 3.4. The Density Comparison of Mixtures

Tests were conducted in order to determine which factors have an impact on the properties of materials. Statistical analysis was used to determine if the factors had a significant effect on the parameters based on the study results. The empirical measurements provided information as to the density of the picture as well as the density of the true picture. The factors that were deemed suitable for statistical analysis were the empirical measurements that had been made. The material type and its kind were deemed quality predictors during the statistical analysis. Statistical analysis of the data from the studies was performed to verify the performance of the program in terms of color percentage in images. A desaturated image was correlated with the X-ray to identify the structure percentage.

With the help of the statistical test between the measured parameters, it is possible to calculate the conversion factor between densities. If the X-ray spectrum is taken into account in conjunction with the true density results, the conversion factor may be of value for preliminary or indicative measurements. In order to identify imaging errors, the conversion factor can be used as a tool. Based on the results of this study, the mean value of the conversion factor for determining 50% PLA in biocomposite was equal to 0.73 and for determining 25% PLA content in biocomposite was equal to 0.603. This indicates that the proposed conversion factor can make materials densities measurement simpler, cheaper, and faster. As an additional benefit of the work, the development of the conversion factor for density measurement was utilized to obtain two different research methods, and this was an added benefit of the work.

## 4. Discussion

In this study, we conducted research to confirm the compatibility of density results obtained through X-ray and photooptical methods for the same samples. This was achieved by performing a comparative experiment. Selected samples underwent both measurement methods, and the results obtained were meticulously analyzed and compared. Upon thorough examination of the data, it was discovered that both X-ray and photooptical methods exhibit a high level of agreement when determining sample densities. Notably, there were no statistically significant differences observed between the density results derived from these two methods.

The present study showcases the substantial impact of biocomposite density with weight proportions of 25% and 50% PLA. The outcomes obtained through X-ray analysis and an innovative technique involving the analysis of reflection from white and dark colors have indicated that the novel photo analysis technology yields results practically equivalent to X-ray measurement technology. After analyzing the outcomes, it was established that the *p*-value was below 0.05, signifying the significance of the obtained results. Specifically, the empirical statistic was computed.

According to various sources in the literature [29], the ash content in wood chips amounts to 1.426%. Since the ash content in PLA material is influenced by its saturation with mineral compounds [5,30], the PLA material is not solely artificial due to its substantial organic macronutrient content. As the PLA content increases, the ash content in the prepared materials diminishes, albeit no significant differences were observed between the two homogenous groups regarding ash content. Furthermore, in line with the literature, such a polymer can also be produced from recyclable sources like corn and potatoes, thereby fostering subsequent utilization within the recycling market [31].

For optical examinations, a microscope was employed to analyze sample structures along with disparities resulting from varying PLA content. Alterations in surface characteristics post-strength testing were also discernible. In instances where the fraction contained 50% PLA instead of 25% PLA, the fraction exhibited enhanced structural organization and reduced roughness. Utilizing image desaturation and PLA content identification, the grayscale density factor was calculated based on PLA proportion within the material. A notable discrepancy between the two homogenous groups was identified in this context. The average density factor for 50% PLA was computed as 0.73, while for 25% PLA, it was 0.66. Consequently, the standard deviation of the density factor for 50% PLA was adopted as the conversion factor. The PLA content demonstrated an increasing variation in the density factor, a trend evident in the statistical plot. Upon organoleptic testing, PLA emitted no unpleasant odors or test-related issues, thereby favoring the possibility of conducting further analyses on biocomposites incorporating this material.

## 5. Conclusions

Confirming the compatibility of X-ray and photooptical density results for identical samples instills a sense of assurance and reliability in both measurement techniques for ascertaining material density. These findings hold promise for future density investigations, potentially yielding heightened precision and comprehensive physicochemical analyses in the realm of science and engineering. In the realm of material density assessment, researchers can confidently utilize both X-ray and photooptical methods, tailoring their selection based on material-specific attributes and available technical resources.

Microscopic examination of the composite structure elucidates PLA’s role in the bonding process. Incorporating PLA additives during biocomposite production can enhance the strength properties, plasticity, and aesthetics of the end. Owing to its biodegradability, PLA proves advantageous for composite formulations, posing no environmental concerns. Furthermore, its future recyclability renders PLA suitable for a diverse array of bio-waste applications. The inclusion of PLA as a binding agent in wood products holds intriguing potential, serving as an adhesive. Notably, PLA could also serve to bind materials presently employed in furniture board fabrication as well as waste generated during furniture usage or modification [32]. With its low hygroscopic nature, PLA stands as an appealing choice for dry environments where minimal interaction with moisture is desirable [33].

In summary, non-invasive methodologies like X-ray or photo-based grayscale analyses can effectively compete. The latter, being significantly more cost-effective and practical in specialized daily use, presents a viable alternative to expensive X-ray methods.

## Figures and Tables

**Table 1:** Influence of the raw material mixture on the density of the tested biocomposite.

PLA Content, %	Position	Density
50	Up	0.720 ^d^
50	Half	0.683 ^c,d^
50	Side	0.777 ^e^
25	Up	0.559 ^a^
25	Half	0.617 ^b^
25	Side	0.633 ^b,c^

^a,b,c,d,e^—homogenous group.

**Table 2:** The biocomposite density profile samples with 50 and 25% PLA content.

PLA Content, %	Sample Name	Sample Dimensions, mm			Weight, g	The Mean Sample Density, kg/m^3^
		Height	Width	Thickness		
50	Sample I	52.04	51.42	7.13	10.115	530.16
	Sample II	50.54	50.95	7.32	11.828	627.51
	Sample III	51.30	50.83	7.21	9.515	506.10
25	Sample I	49.32	51.16	7.12	10.402	579.01
	Sample II	50.11	52.41	7.01	10.172	552.52
	Sample III	49.2	50.12	7.29	10.369	576.81

## Data Availability

Not applicable.
